# A Rare Case of Headache in a Patient With McCune-Albright Syndrome: A Triple Threat

**DOI:** 10.7759/cureus.45249

**Published:** 2023-09-14

**Authors:** Ravi Shah, Liza Das, Pinaki Dutta, Ashwani Sood, Sanjay Kumar Bhadada

**Affiliations:** 1 Endocrinology, Postgraduate Institute of Medical Education and Research, Chandigarh, IND; 2 Nuclear Medicine, Postgraduate Institute of Medical Education and Research, Chandigarh, IND

**Keywords:** mccune-albright syndrome, headache, fibrous dysplasia, tuberculosis, acromegaly

## Abstract

A 26-year-old male presented with facial asymmetry since 11 years of age and painless progressive diminution of vision in the left eye since 16 years of age. He presented with an exacerbation of headaches for the past two months. On examination, he was tall and had acral enlargement, craniofacial deformity, and bilateral asymmetric testicular enlargement. Investigations revealed high insulin-like growth factor 1, non-suppressible growth hormone on oral glucose tolerance tests, and multiple pituitary hormone deficiencies. MRI showed pituitary macroadenoma with craniofacial and sphenoid fibrous dysplasia as well as multiple tuberculomas. Cerebrospinal fluid testing showed high protein, low glucose, and high adenosine deaminase, all consistent with a diagnosis of central nervous system (CNS) tuberculosis. His headache did not respond significantly to either octreotide or zoledronic acid. The patient was then initiated on antitubercular therapy, which led to near-complete resolution of the headache and CNS lesions within three months of therapy. CNS tuberculosis was a masquerader in the index case of acrogigantism due to McCune-Albright syndrome. Headaches may be multifactorial in a given case of acromegaly, and investigating for alternative or additional causes especially when dealing with treatment-refractory cases can be rewarding.

## Introduction

McCune-Albright syndrome (MAS) is a rare disorder characterized by post-zygotic somatic mutation of the *GNAS *gene leading to mosaic activation of Gs alpha in different tissues. This syndrome was first described by Dr. McCune and Dr. Albright as a classic triad of polyostotic fibrous dysplasia, café-au-lait macules, and precocious puberty [1]. On retrospective review, a similar history was found in Thomas Hasler, the “Tegernsee Giant,” who was reported to be a normal developing boy till nine years of age when he developed a height spurt with enlargement of the skull and fracture of the left-sided fibula [2]. He was likely the first reported case of MAS in retrospect. It can have a varied presentation with the extent of involvement of any part of the skeleton along with cutaneous features in the form of café-au-lait macules, endocrine involvement in the form of precocious puberty, non-autoimmune hyperthyroidism, acromegaly, and neonatal hypercortisolism. Acromegaly affects about 20% of patients with MAS [3].

Here, we describe a case of acrogigantism associated with MAS with a severe headache not responding to medical management with octreotide as well as zoledronic acid. Further evaluation revealed central nervous system (CNS) tuberculosis, the third and, probably, the primary contributor to headache in the index patient other than acromegaly and fibrous dysplasia.

## Case presentation

**Figure 1:**
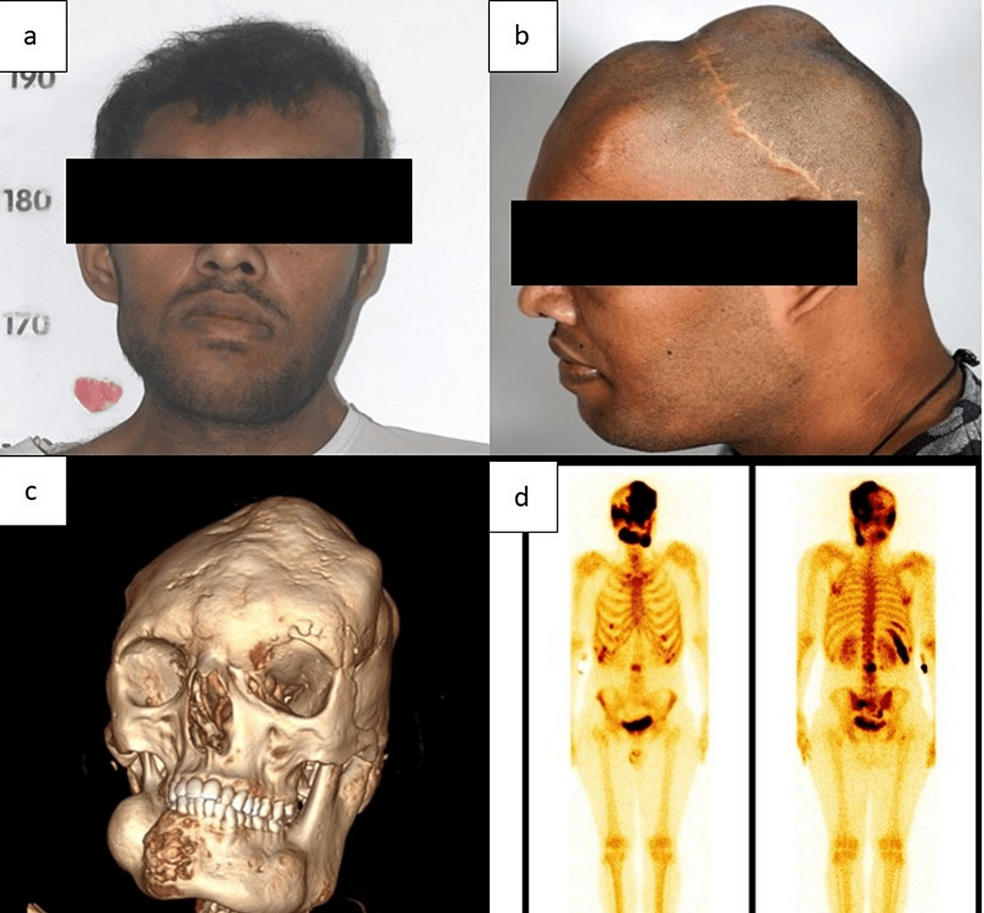
Clinical image of the patient showing acrogigantism (height 192 cm) (a) and irregular bumpy skull vault consistent with craniofacial fibrous dysplasia and acanthosis nigricans in the nape of the neck (b). Three-dimensional CT reconstruction of facial skeleton showing facial asymmetry (c) and whole body Tc99 methylene diphosphonate skeletal scintigraphy showing increased osteoblastic activity in skull bones, maxilla, both sides of the mandible, multiple bilateral ribs, L3 vertebra, and sacrum with bilateral ala suggestive of polyostotic fibrous dysplasia (d).

A 26-year-old male presented with a headache along with profuse sweating for two months before the presentation. The headache did not show diurnal variation and was graded as 9/10 on the visual analog scale. The patient had noticed acral enlargement over the last 10 years, with an increase in shoe size from 8 to 11 (UK size), facial asymmetry since 11 years of age, and painless progressive diminution of vision in the left eye since 16 years of age. There was a complete loss of vision in the left eye since he was 18 years of age, which subsequently progressed to an inability to notice objects in his right visual field. There was no history suggestive of precocity. On examination, he was tall (height = 192 cm) with a height standard deviation score (SDS) of +2.76 SDS and mid-parental height adjusted height SDS of +2.38 SDS, suggestive of acrogigantism. He had coarsening facial features with thick lips, spaced-out teeth, facial and skull asymmetry (Figures 1, Panels a-c), acral enlargement, and a sonorous voice. He also had sweaty palms. Visual examination revealed complete loss of vision in the left eye with optic atrophy and right temporal hemianopia on perimetry. He also had bilateral testicular enlargement (bilateral testis >25 mL) by Prader’s orchidometer. The left testis was also stony hard in consistency. There were no café-au-lait macules. The family history was not significant. Investigations revealed high insulin-like growth factor 1 (IGF1) (457 ng/mL, reference range (RR) = 130-295 ng/mL) and non-suppressible growth hormone (GH) on oral glucose tolerance test (nadir GH = 26 ng/mL), consistent with active acromegaly. Other baseline investigations are summarized in Table 1.

**Table 1:** Biochemical and hormonal profile at presentation.

Parameter	Patient value	Reference range
Hemoglobin (g/dL)	11.5	>12
Creatinine (mg/dL)	0.82	0.5–1.2
Alanine transaminase/Aspartate transmainase (U/L)	42/40	2–41/2–40
Serum calcium (mg/dL)	8.52	8.6–10.2
Albumin (g/dL)	3.9	3.4–4.8
Serum phosphorus (mg/dL)	3.88	2.5–4.5
Alkaline phosphatase (IU/L)	309	42–128
25(OH) vitamin D (ng/mL)	5.83	30–100
Intact parathyroid hormone (pg/mL)	59	15–65
Basal growth hormone (ng/mL)	45.7	0–2.5
Nadir growth hormone on glucose tolerance test (ng/mL)	26	<0.4
Insulin-like growth factor 1 (ng/mL)	457	130–295
Prolactin (ng/mL)	28.4	4.79–23.3
Testosterone (ng/mL)	0.42	2.8–8.1
Total T4 (µg/dL)	4.98	4.8–12.7
Cortisol (ng/mL)	11.45	61–195
Procollagen-1 intact N-terminal propetide (ng/mL)	432	15–58
Beta cross laps (pg/mL)	1107	16–584
Serum *luteinizing hormone* (mIU/mL)	0.3	1.7–8.6
Serum follicle-stimulating hormone (mIU/mL)	1.41	1.5–12.4
Serum thyroid-stimulating hormone (µIU/mL)	1.31	0.27–4.2
Serum T3 (ng/mL)	1.6	0.8–2

**Figure 2:**
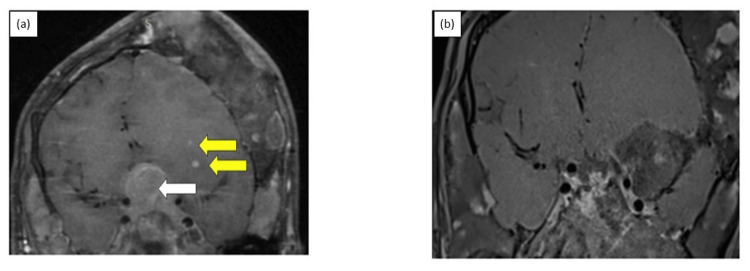
Preoperative post-contrast T1-weighted MRI sella and brain suggestive of (a) pituitary macroadenoma (white arrow) with multiple ring-enhancing lesions (yellow arrows) with (b) resolution of ring-enhancing lesion post-antitubercular therapy and decrease in the size of adenoma post-transsphenoidal surgery.

The patient was prescribed subcutaneous short-acting injectable octreotide (100 µg) thrice daily while awaiting imaging confirmation. Polyostotic fibrous dysplasia was suspected bearing in mind the facial asymmetry and acro gigantism and a Tc-99 whole-body methylene diphosphonate scan was performed, which confirmed the same (Figure 1, Panel d). As the patient had headaches not responding to octreotide, he was given injectable zoledronic acid, but again with no significant relief in symptoms. For evaluation of acromegaly, he underwent contrast-enhanced MRI (CEMRI) of the sella and brain which revealed a pituitary macroadenoma measuring 2.1 x 3.6 x 2.3 cm with optic chiasma compression and bilateral parasellar extension with maintained flow voids in bilateral internal carotid artery (Figure 2, white arrow). Incidentally, the scan also showed multiple ring-enhancing lesions throughout the brain parenchyma (Figure 2, yellow arrow). A diagnosis of CNS tuberculosis with tuberculomas was contemplated, and lumbar puncture showed lymphocytic pleocytosis with high protein (532 mg/dL; RR = 15-60 mg/dL), low glucose (32 mg/dL; RR = 50-80 mg/dL), and elevated adenosine deaminase value (42 IU/L RR = <10 IU/L), favoring CNS tuberculosis. The cerebrospinal fluid polymerase chain reaction for *Mycobacterium tuberculosis* was negative.

For the evaluation of testicular enlargement, serum alpha-fetoprotein, human chorionic gonadotrophin, and lactate dehydrogenase were tested and were found to be within normal limits. Serum anti-Mullerian hormone was low (0.2 ng/mL; RR = 3-18 ng/mL), pointing toward testicular failure. Ultrasonography of the testes revealed bulky, heterogenous left testis and bilateral epididymis with nodular areas of enhancement in left testes, likely epididymal-orchitis with granulomatous lesions due to tuberculosis, and these findings were corroborated on MRI of the scrotum. Chest X-ray was suggestive of hilar pulmonary infiltrates likely secondary to tuberculosis. Hence, a diagnosis of disseminated tuberculosis was made.

The patient was then initiated on antitubercular therapy (ATT) and dexamethasone. Transcranial surgery was delayed given the diagnosis of CNS tuberculosis and planned following the completion of the intensive phase of ATT. He was also offered monthly long-acting release injectable octreotide long-acting release of 20 mg for symptomatic benefit. The patient was also initiated on hormone replacement for corresponding multiple pituitary hormone deficiency (Table 1).

After the patient completed the intensive phase of ATT (two months), he was re-evaluated and found to have a significant reduction in the intensity of headache with a visual analog scale reducing to 5/10. Basal GH on reassessment was 31.5 ng/mL with serum IGF1 increasing to 797 ng/mL. He then underwent endoscopic transsphenoidal surgery, with histopathology suggestive of the pituitary neuroendocrine tumor with Ki67 of 5-6%. Immediate postoperative random GH was 12.5 ng/mL. The patient was continued on injectable octreotide long-acting release of 20 mg monthly. Despite six months of octreotide therapy, there was no reduction in IGF1 with basal GH 6.55 ng/mL, which was non-suppressible on the glucose tolerance test (3.4 ng/mL). Repeat CEMRI revealed a significant decrease in the size of the pituitary tumor to 1.5 x 3.6 x 1.1 cm with a 66% reduction in volume with no evidence of ring-enhancing lesions or meningeal enhancement on imaging (Figure 2). Even though the patient did not undergo biochemical remission, he had almost complete resolution of headache with just ATT, graded as 1/10 by the patient on the visual analog scale. There was also a 60% reduction in the volume of both testes with ATT and the disappearance of pulmonary infiltrates. Given persistent and active acromegaly, the patient was planned for radiotherapy after a decision by a multidisciplinary meeting to follow up with the continuation of octreotide in the interim.

## Discussion

A literature review was performed to identify previously reported cases of acromegaly and sellar or extracellular tuberculosis (Table 2). In the last 15 years, we have encountered 321 cases of acromegaly who underwent transsphenoidal surgery at our institute, with five patients having concomitant tuberculosis at the time of diagnosis. They were probably due to chance association. These details are also mentioned in Table 2.

**Table 2:** A literature review of cases of acromegaly described in association with tuberculosis.

Case	Age/Sex	Presenting feature	Final Diagnosis
Ellman et al. [5]	59/M	Acromegaly features with hemoptysis	Acromegaly (pituitary macroadenoma) with pulmonary tuberculosis
Bhadada et al, [6]	24/F	Acromegaly features, galactorrhea, and abdominal pain	Acromegaly (pituitary adenoma) with intrasellar tuberculoma and abdominal tuberculosis
Sharma et al. [7]	24/F	Acromegaly features with headache	Pituitary adenoma with intrasellar tuberculoma
Present case	26/M	Acrogigantism with craniofacial fibrous dysplasia presenting with headache	McCune-Albright syndrome with acrogigantism due to a pituitary macroadenoma with polyostotic fibrous dysplasia, concurrent cranial tuberculosis, pulmonary tuberculosis, and tubercular epididymal-orchitis

To our knowledge, the index case is the first report of the association of CNS tuberculosis with acromegaly and polyostotic fibrous dysplasia in literature. Headache in the index patient was multifactorial, attributable to acromegaly due to a pituitary macroadenoma, fibrous dysplasia of the craniofacial region, and CNS tuberculosis. However, the therapeutic response of the patient to ATT but neither octreotide for acromegaly nor zoledronic acid for polyostotic fibrous dysplasia suggested that CNS tuberculosis was the predominant contributor to his headache.

CNS tuberculosis accounts for 20% of intracranial space-occupying lesions in India and responds quite well to ATT [4]. The association of acromegaly with concurrent CNS tuberculosis is rare, with only three cases in the literature described hitherto (Table 2) [5-7]. Our case is unique in being the first to report the association of fibrous dysplasia and acrogigantism (MAS) with extrasellar but intracranial tuberculosis and describe the distinct diagnostic and therapeutic challenges.

Headache in MAS is multifactorial and is generally caused by direct GH action of GH-secreting pituitary adenoma causing calvarial thickening, the mass effect of the pituitary adenoma, craniofacial fibrous dysplasia, or a combination of any of these. However, our patient did not respond to either octreotide or zoledronic acid, and an MRI of the sella and brain with contrast revealed CNS tuberculosis. Following ATT, his headache responded and showed near-complete resolution. This was further ratified by the fact that the patient had persistent acromegaly and a residual tumor following transsphenoidal surgery, but his headache did not persist.

The testicular enlargement in the index patient was initially attributed to MAS owing to a high prevalence of testicular involvement in MAS (reported in approximately 85% of patients). Histopathology in these cases generally shows Leydig and Sertoli cell hyperplasia, with Sertoli cell hyperplasia being more common [3]. Autonomous testosterone production is less common (reported in up to 15% of cases), and it was not present in the index patient. In our patient, testicular enlargement was initially attributed to the above reason, but imaging and response to ATT suggested tubercular epididymal-orchitis.

## Conclusions

Headache in MAS is often severe and usually caused by pituitary mass and craniofacial fibrous dysplasia. This case highlights how CNS tuberculosis can confuse the clinical presentation in a case of MAS, warranting a closer look at the subset of patients who remain treatment-refractory for the evaluation of alternative and additional causes.
